# The thickness change ratio and preservation ratio of the infrapatellar fat pad are related to anterior knee pain in patients following medial patellofemoral ligament reconstruction

**DOI:** 10.1186/s13018-024-04853-2

**Published:** 2024-06-25

**Authors:** Zhenhui Huo, Chenyue Xu, Sibo Li, Yingzhen Niu, Fei Wang

**Affiliations:** 1 Department of Orthopaedic Surgery, Third Hospital of Hebei Medical University, Shijiazhuang, 050051 Hebei China; 2 School of Pharmacy, Hebei University of Chinese Medicine, Shijiazhuang, 050200 Hebei China

**Keywords:** Patellar dislocation, Infrapatellar fat pad, Anterior knee pain, Medial patellofemoral ligament reconstruction

## Abstract

**Background:**

The infrapatellar fat pad (IPFP) lies extrasynovial and intracapsular, preserving the joint cavity and serving as a biochemical regulator of inflammatory reactions. However, there is a lack of research on the relationship between anterior knee pain (AKP) and the IPFP after medial patellofemoral ligament reconstruction (MPFLR). Pinpointing the source of pain enables clinicians to promptly manage and intervene, facilitating personalized rehabilitation and improving patient prognosis.

**Methods:**

A total of 181 patients were included in the study. These patients were divided into the AKP group (*n* = 37) and the control group (*n* = 144). Clinical outcomes included three pain-related scores, Tegner activity score, patient satisfaction, etc. Imaging outcomes included the IPFP thickness, IPFP fibrosis, and the IPFP thickness change and preservation ratio. Multivariate analysis was used to determine the independent factors associated with AKP. Finally, the correlation between independent factors and three pain-related scores was analyzed to verify the results.

**Results:**

The control group had better postoperative pain-related scores and Tegner activity score than the AKP group (*P* < 0.01). The AKP group had lower IPFP thickness change ratio and preservation ratio (*P* < 0.001), and smaller IPFP thickness (*P* < 0.05). The multivariate analysis revealed that the IPFP thickness change ratio [OR = 0.895, *P* < 0.001] and the IPFP preservation ratio [OR = 0.389, *P* < 0.001] were independent factors related to AKP, with a significant correlation between these factors and pain-related scores [|r| > 0.50, *P* < 0.01].

**Conclusions:**

This study showed the lower IPFP change ratio and preservation ratio may be independent factors associated with AKP after MPFLR. Early detection and targeted intervention of the underlying pain sources can pave the way for tailored rehabilitation programs and improved surgical outcomes.

**Level of evidence Level III:**

## Introduction

Patellar dislocation (PD) is a common causes of knee injuries in adolescents; the incidence of PD ranges from 29 to 43 per 100,000 per year [1, 2]. The main risk of recurrent PD is patellofemoral instability, but additional complications like chondral or osteochondral damage and decreased knee function may also occur [3, 4].

Biomechanical studies confirm the medial patellofemoral ligament (MPFL) is the primary medial stabilizer of the patella, providing 50–60% of restraining force within the initial 0° to 30° of knee flexion [5, 6]. The inadequacy or damage to the MPFL is a significant risk factor for PD [7]. Following a precise diagnosis of PD through physical examination, scoring systems, and imaging data [8], MPFL reconstruction (MPFLR) has emerged as the primary treatment method, delivering satisfactory clinical outcomes, favorable improvements in functional outcomes and low complication rates, even in patients with bone deformities [9, 10].

Despite favorable outcomes, MPFLR is not exempt from complications. Anterior knee pain (AKP) is among the most commonly encountered morbidities associated with MPFLR, with an estimated incidence ranging from 0 to 32.3% [11]. AKP has been shown to reduce activities of daily living and physical activity [12] and may disappoint patients with surgical treatment. Adolescent patients have high expectations for postoperative exercise recovery, but AKP can negatively impact their prognosis and confidence.

The infrapatellar fat pad (IPFP), composed of somatic adipose tissue, lies extrasynovially and intracapsular. It fills empty space within the joint, preserving the joint cavity, facilitating effective lubrication, and acting as a biochemical regulator of inflammatory and destructive reactions in the injured knee [13, 14].

The relationship between AKP and the IPFP in patients with osteoarthritis (OA) and anterior cruciate ligament (ACL) reconstruction has been widely studied, and there is a strong correlation between AKP and the IPFP [15, 16]. The IPFP serves both biomechanical and inflammatory functions in the patellofemoral joint, secreting cytokines associated with AKP [17]. The IPFP, akin to the synovium, is characterized by abundant vascularization and nociceptor nerve fiber innervation. Post-joint trauma, inflammation in the synovial membrane and IPFP often leads to soft tissue impingement [18]. This impingement can trigger further inflammation, creating a harmful cycle of IPFP hypertrophy, tissue impingement, knee pain, and release of inflammatory compounds [19, 20]. This cycle may contribute to AKP in patients.

However, the association between the IPFP and the incidence of AKP after MPFLR is still not fully understood. Identifying the source of pain could help clinicians manage and intervene early, which is beneficial to the individualized rehabilitation and good prognosis of patients. The purpose of this study was to identify the independent factors associated with AKP after MPFLR through magnetic resonance imaging (MRI) and ultrasound (US) dynamic evaluation. It was hypothesized that the lower IPFP thickness change ratio and the lower IPFP preservation ratio may be independent factors associated with AKP.

## Materials and methods

### Patient recruitment

Institutional review board approval was acquired from the ethics committee of the Third Hospital of Hebei Medical University (No. Ke2023-002-1), and informed consent was obtained from all patients before the initiation of this retrospective study.

The medical records of patients with unilateral recurrent PD who underwent MPFLR between January 2017 and January 2023 were identified and reviewed. The inclusion criteria were as follows: (1) two or more episodes of PD; (2) a history of PD with symptoms of patellar instability (pain, subluxation, or both) for more than 3 months; (3) unilateral PD; (4) skeletal maturity; (5) at least 12 months follow-up; (6) a positive patellar apprehension sign; and (7) conservative treatment was ineffective. The exclusion criteria were as follows: (1) traumatic or habitual dislocation; (2) previous knee surgery patients; (3) generalized joint laxity; (4) concomitant ligament reconstruction (cruciate ligament or collateral ligament); (5) revision cases; (6) missing clinical data; (7) *Q* angle > 20°; (8) tibial tuberosity-trochlear groove distance > 20 mm; (9) rotational malalignment (femoral anteversion or tibial external torsion > 30°); (10) patella alta with Caton-Deschamps index > 1.2; (11) high-grade trochlear dysplasia (grades B, C or D of Dejour’s classification [21]); and (12) trochlear angle > 145°. Patients with patellofemoral arthritis, rheumatoid arthritis, osteonecrosis, neurological disorders, or medical conditions that affected neuromuscular function were excluded as well.

**Fig. 1:**
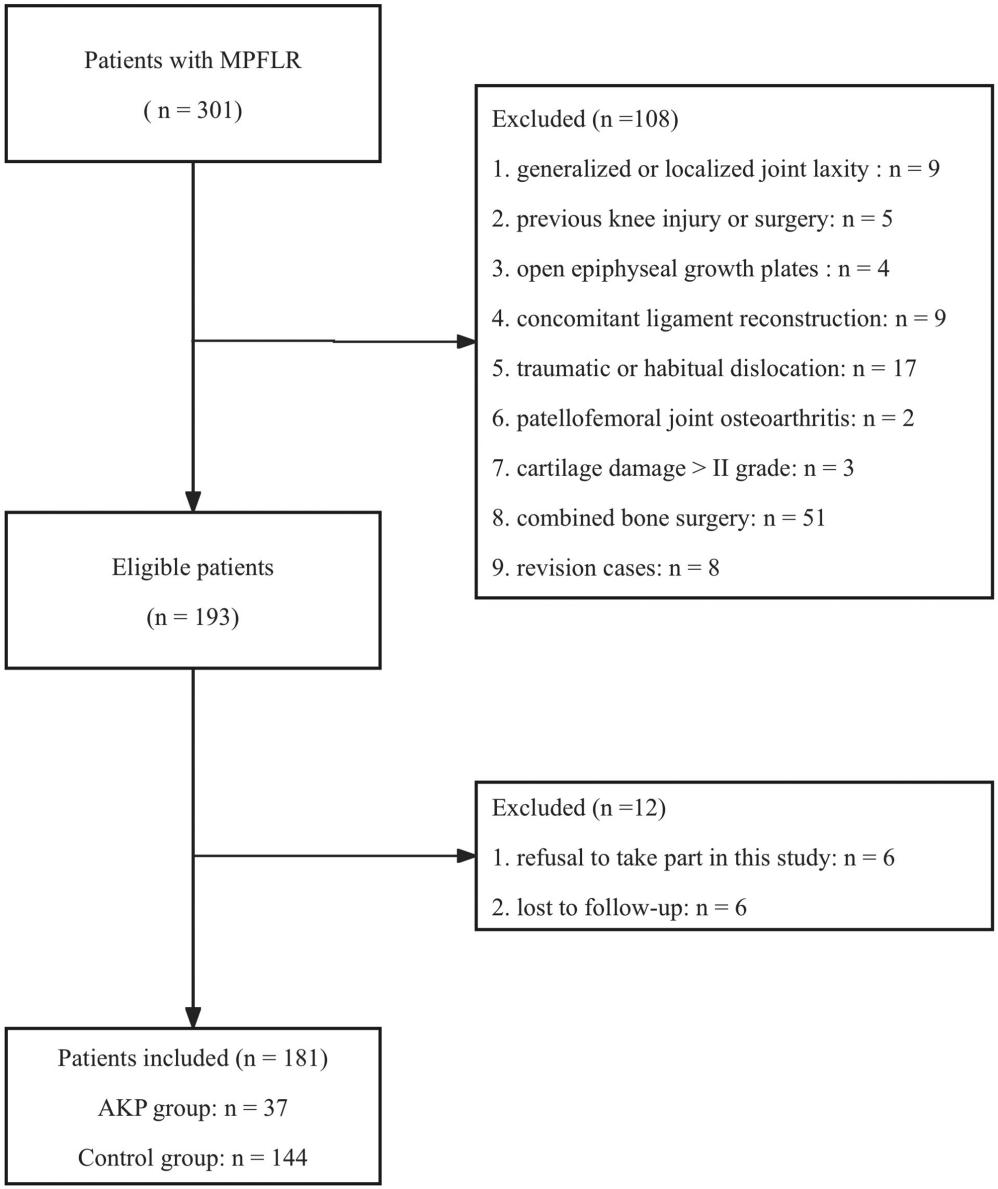
Flowchart of the patient selection. *MPFLR* medial patellofemoral ligament reconstruction, *AKP* anterior knee pain

Based on the abovementioned criteria, from a total of 301 screened patients, 187 patients were included. During the postoperative outpatient visit, after reaching a stable state of recovery (at least 12 months), the surgeon evaluated AKP. The anterior region of the patient’s knee was pointed out and explained by the same clinical surgeon. These patients were divided into two groups: the AKP group and control group. Patients in the AKP group should meet the following conditions: (1) Discomfort must persist for at least two weeks with one of the following: [22]: (i) prolonged sitting; (ii) stair walking; (iii) patella compression; or (iv) quadriceps isometric contraction (in a seated position with knee extended); (2) The Kujala score evaluated symptoms during activities associated with AKP: walking, running, jumping, climbing stairs, squatting, and sitting with the knee bent [23, 24]. Responses are categorized as either “no difficulty” or “other,” with patients selecting “other” indicating the presence of anterior knee symptoms [25]. (3) Patients with an numerical rating scale (NRS) score of ≥ 1 point were considered to have anterior knee symptoms [26]. Patients with AKP who met the above three conditions were classified as the AKP group (*n* = 39), and other patients were classified as the control group (*n* = 148). All surgeries were performed by the same senior surgeon. Six patients (2 in the AKP group and 4 in the control group) were lost to follow-up, resulting in 37 patients in the AKP group and 144 patients in the control group with a minimum 12-month follow-up period (Fig. 1).

### Surgical procedure and postoperative rehabilitation

All surgeries were performed by the same senior surgeon. Double-bundle anatomic MPFLR was performed using the ipsilateral gracilis tendon. The femoral tunnel was positioned with reference to the osseous landmarks between the medial femoral epicondyle and the adductor tubercle and was verified with fluoroscopy according to the method described by Schöttle et al. [27]. Two patellar tunnels were drilled in the upper corner and the center of the medial edge of the patella. Three ends of the graft were fixed with absorbable screws. After fixation of the patella end, the patellar tracking, graft tension, and the lateral retinacula tightness were checked by arthroscopy in extension and flexion. If the patella was stable, the femoral end would be fixed.

All patients followed the standard phased rehabilitation program. The quadriceps strengthening exercise began immediately after the patient’s tolerance [28]. Non-weight-bearing was allowed for the first 3 weeks after surgery, and long hinge knee braces were used to protect the knee joint, allowing passive motion of 0° to 30°. The flexion was allowed to progress to 90° in 3–6 weeks, and partial weight-bearing was allowed within the patient’s tolerance range. The knee brace could be removed at 6 weeks, allowing patients to perform active knee joint activities and full weight-bearing exercises. Patients were encouraged to return to physical activity around 6 months after surgery.

### Evaluation methods

#### Clinical evaluation

Demographic data, including age, sex, time from injury to surgery and body mass index (BMI) were collected, and postoperative follow-up records were reviewed. All clinical evaluations were collected before surgery and at the last follow-up. The Kujala, Visual Analog Scale (VAS), and the NRS scores as pain-related scores are frequently used functional scales to assess pain [23, 29]. The activity level was measured with the Tegner activity score [30]. The recurrence of subluxation or dislocation was documented. Satisfaction was evaluated using four levels: very satisfied, satisfied, partially satisfied, and not satisfied at all.

### Imaging evaluation

#### US examination and measurements

**Fig. 2:**
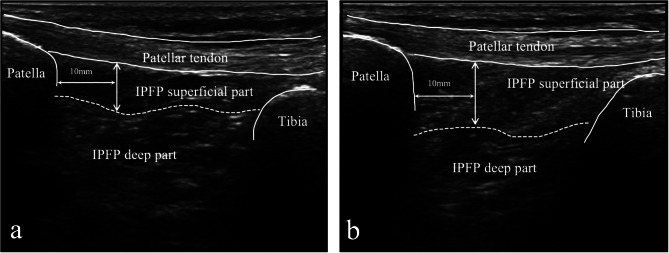
The measurement of the IPFP superficial part measurement at a 10° (**a**) and 90° (**b**) knee flexion. *IPFP* infrapatellar fat pad

According to the previous method of Kitagawa et al. [31, 32], participants were placed in a sitting position to capture ultrasonography. The thickness of the superficial part of the IPFP was assessed on rebuilt knees at 10° or 90° knee flexion using a goniometer, and longitudinally oriented ultrasonographic images of the anterior part of the knees were taken at the center of the patellar tendon. A low-echo intensity area above the high-intensity septum in the middle region of the IPFP was designated as the superficial part of the IPFP. The IPFP thickness was measured 10 mm away from the patellar apex (Fig. 2). Given the dynamics of the IPFP during knee motion, the ratio of the change in thickness of the IPFP between the two flexion angles was calculated using the following formula: the IPFP thickness change ratio = (the thickness of the superficial part of the IPFP at 90° knee flexion) / (the thickness of the superficial part of the IPFP at 10° knee flexion) [32].

#### MRI technique and measurements

**Fig. 3:**
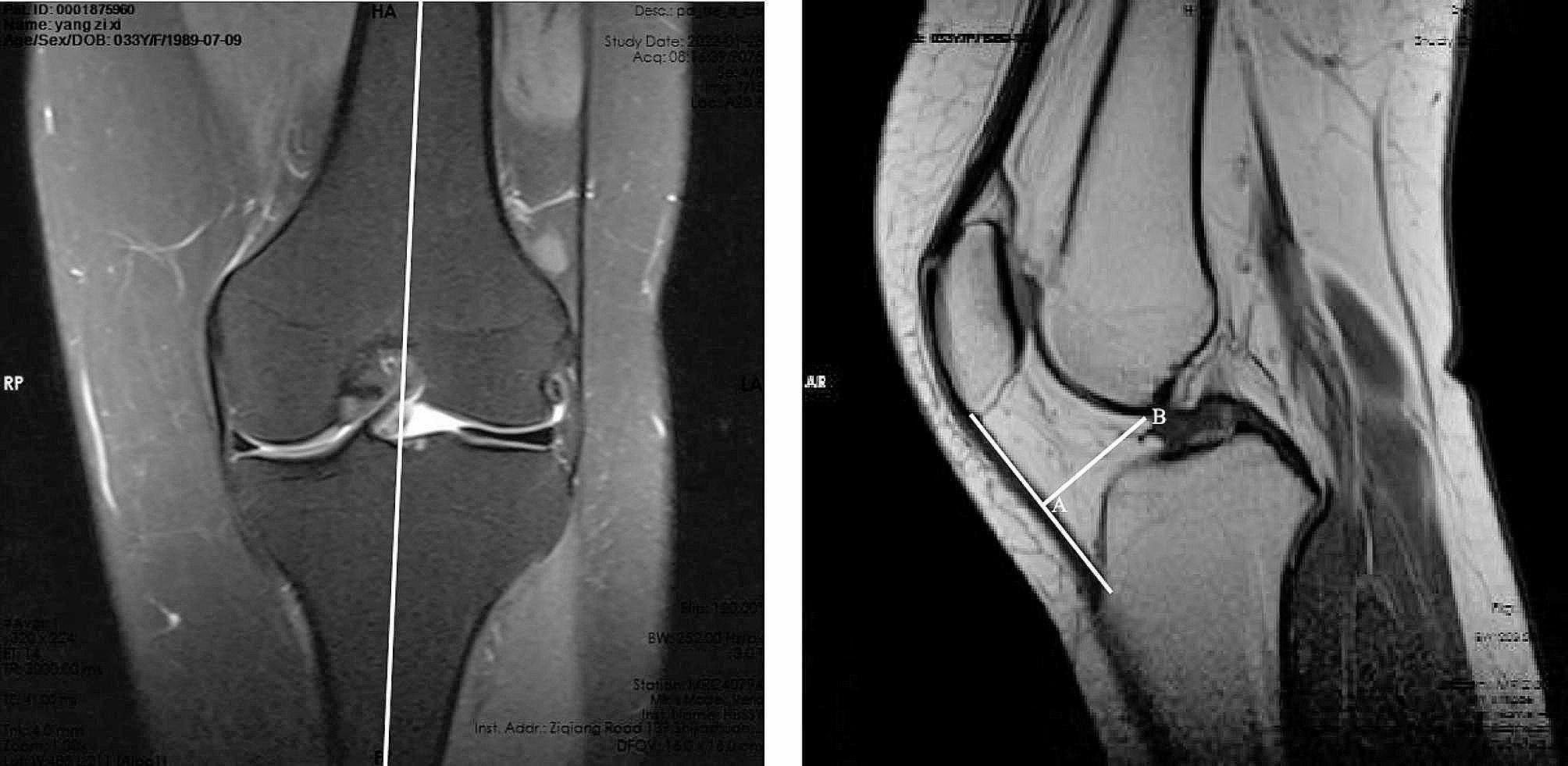
The measurement of the IPFP thickness. A baseline was drawn on the leading edge of the IPFP, and the distance (AB) between it and the farthest point on the posterior edge was taken as the thickness of the IPFP. *IPFP* infrapatellar fat pad

**Fig. 4:**
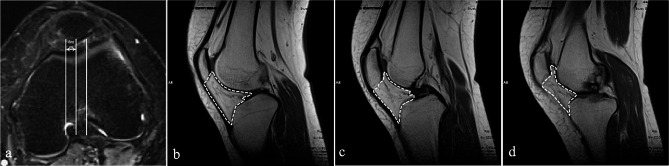
The calculation of the IPFP size. **a** Sagittal images at the three locations on the axial plane that were used. **b, c,d** The total IPFP size on three sagittal planes. *IPFP* infrapatellar fat pad

The method of Giovanni Ricatti et al. [33] was improved to measure the IPFP thickness. The sagittal image close to the midpoint of the knee joint and perpendicular to the articular surface was selected as the measurement image. A baseline was drawn on the leading edge of the IPFP, and the distance between it and the farthest point on the posterior edge was taken as the thickness of the IPFP (Fig. 3). The total size of the IPFP was determined by analyzing sagittal MRI at three specific locations on the axial plane: the deepest, medial, and lateral portions, 6 mm from the deepest point of the femoral trochlear groove. The IPFP preservation ratio was defined as the rate of the total IPFP size at the last follow-up postoperatively compared to that preoperatively [34] (Fig. 4). In addition, fibrosis of the IPFP was also evaluated. IPFP fibrosis was characterized by areas of low intensity in the IPFP on both T1- and T2-weighted images during the last follow-up after MPFLR, which were not present before the operation [35].

### Data measurement

All evaluations were conducted based on US images obtained at the last follow-up, as well as MRI images acquired within 1 week before surgery and at the last follow-up. These indicators encompassed the IPFP thickness change ratio, the IPFP preservation ratio, as well as the IPFP thickness and IPFP fibrosis. All indicators were made independently by two experienced surgeons twice using the same criteria at a 2-week interval, and the means of the data were taken as the final results for analysis. The interobserver and intraobserver agreement of the indicators was calculated. The intraclass correlation coefficient (ICC) values with 95% confidence intervals were calculated, and an ICC value > 0.8 indicated excellent reliability. The interobserver and intraobserver reliability of the measurement was shown in Table 1.

**Table 1:** The inter- and intraobserver reliability of different measurements

Variable	Interobserver reliability	Intraobserver reliability
The IPFP thickness	0.87 (0.81–0.91)	0.92 (0.86–0.94)
The IPFP thickness change radio	0.92 (0.86–0.93)	0.89 (0.87–0.91)
The IPFP preservation ratio	0.86 (0.84–0.90)	0.92 (0.88–0.94)
IPFP fibrosis	0.94 (0.90–0.96)	0.96 (0.93–0.97)

*IPFP* infrapatellar fat pad

### Statistical analysis

The data were analyzed using IBM SPSS version 26.0 (SPSS Inc., Chicago, IL, USA) software. The Kolmogorov‒Smirnov test was used to determine the normality of the data. The analysis was performed with the two-tailed Student’s t test for data with a normal distribution (e.g., age, BMI and follow-up duration) or the Mann‒Whitney U test for nonparametric data (e.g., the IPFP thickness and change ratios). Categorical variables were compared by the Chi-square test (e.g., gender, side, and IPFP fibrosis). The data were expressed as the means and standard deviations for continuous variables, and as numbers and percentages for categorical variables. *P* < 0.05 was defined as significant.

The parameters with significant differences between the two groups were included in the multivariate logistic regression analysis. Correlations between three pain-related scores (the Kujala, NRS, and VAS scores) and independent factors associated with AKP were examined using Spearman’s rank-correlation coefficient (r).

A priori power analysis was performed based on the postoperative Kujala score between the two groups. A sample size of 29 patients per group was required for a confidence level of 95% (α = 0.05) and power of 80%.

## Results

In this study, 181 patients who underwent MPFLR and completed preoperative and postoperative standard questionnaire surveys were included, with 20.4% (37 patients) developing symptoms of AKP. Demographic comparison revealed a significantly higher BMI in the AKP group compared to the control group (21.9 ± 3.6 vs. 20.7 ± 3.5, *P* < 0.05). There was no significant difference observed in gender, age, side of the knee, or time from injury to surgery (Table 2).

**Table 2:** Demographic characteristics of the AKP group and the control group

Variable	AKP group	control group	*P* value
Number of patients	37	144	-
Number of knees	37	144	-
Gender (male/female)	6 (16.2%) /31 (83.8%)	42 (29.2)/102 (70.8%)	n.s
Side (left/right)	17 (45.9%)/20 (54.1%)	56 (38.9)/88 (61.1%)	n.s
Age (year)	21.6 ± 9.1	20.2 ± 8.9	n.s
BMI (kg/m^2^)	21.9 ± 3.6	20.7 ± 3.5	< 0.05
Time from injury to surgery (year)	4.7 ± 2.7	4.7 ± 1.2	n.s
Follow-up (month)	14.5 ± 2.1	13.8 ± 1.6	n.s

Data are expressed as n (percentage) or mean ± standard deviation

*AKP* anterior knee pain, *BMI* body mass index, *n.s.* non-significant

At the last follow-up, MRI showed that all patients had good patellofemoral joint alignment, complete grafts, and good fixation, and no patients in either group had experienced dislocation again. Wound infection, deep venous thrombosis of the lower extremities, and continued bone surgery were not found.

Table 3 presented the clinical outcomes of both groups. The control group exhibited significantly higher postoperative scores compared to the AKP group (*P* < 0.001). The Tegner activity score did not significantly improved after operation in the AKP group, and two patients were not satisfied (*P* < 0.05); the percentage of satisfied patients in the other three groups in the control group was higher than that in the AKP group, but the difference was not significant (Table 4).

**Table 3:** Comparison of clinical outcomes between the AKP group and the control group

Variable	AKP group	control group	*P* value
Kujala score			
Preoperative	54.2 ± 6.8	53.0 ± 7.7	n.s
Postoperative	80.3 ± 5.8	90.8 ± 5.6	< 0.001
*P* value	< 0.001	< 0.001	
NRS score			
Preoperative	6.1 ± 0.8	6.2 ± 1.1	n.s
Postoperative	3.0 ± 1.1	0.2 ± 0.1	< 0.001
*P* value	< 0.001	< 0.001	
VAS score			
Preoperative	5.7 ± 0.5	5.5 ± 0.8	n.s
Postoperative	3.7 ± 0.6	1.0 ± 0.6	< 0.001
*P* value	< 0.001	< 0.001	
Tegner activity score			
Preoperative	4 (2–5)	4 (2–5)	n.s
Postoperative	4 (2–6)	5.5 (3–8)	< 0.01
*P* value	n.s	< 0.001	

Data are expressed as mean ± standard deviation or median (range)

*AKP* anterior knee pain, *NRS* Numerical Rating Scale, *VAS* Visual Analog Scale, *n.s.* non-significant

**Table 4:** Patient satisfaction between the AKP group and the control group

Patient satisfaction	AKP group	control group	*P* value
Very satisfied	29 (78.4%)	118 (81.9%)	n.s
Satisfied	3 (8.1%)	18 (12.5%)	n.s
Partially satisfied	3 (8.1%)	8 (5.6%)	n.s
Not satisfied	2 (5.4%)	0 (0%)	< 0.05

Data are expressed as n (percentage)

*AKP* anterior knee pain, *n.s.* non-significant

As presented in Table 5, the postoperative imaging outcomes of the two groups were significantly different. The IPFP preservation ratio (81.0 ± 5.2% vs. 89.6 ± 2.9%, *P* < 0.001), the thickness change ratio (197.0 ± 17.5% vs. 252.1 ± 27.4%, *P* < 0.001), and the IPFP thickness (22.2 ± 1.3 mm vs. 27.5 ± 1.3 mm, *P* < 0.05) in the AKP group were significantly lower than those in the control group. No significant difference in IPFP fibrosis was observed between the AKP group (24.3%, 9/37) and the control group (20.8%, 30/144).

**Table 5:** Comparison of imaging outcomes between the AKP group and the control group

Variable	AKP group	control group	*P* value
The IPFP thickness (mm)	22.2 ± 1.3	27.5 ± 1.3	< 0.05
The IPFP thickness change radio (%)	197.0 ± 17.5	252.1 ± 27.4	< 0.001
The IPFP preservation ratio (%)	81.0 ± 5.2	89.6 ± 2.9	< 0.001
IPFP fibrosis	9 (24.3%)	34 (23.6%)	n.s

Data are expressed as mean ± standard deviation or n (percentage)

*AKP* anterior knee pain, *IPFP* infrapatellar fat pad, *n.s.* non-significant

In the multivariate logistic regression analysis, patients with the lower IPFP preservation ratio [OR = 0.389, *P* < 0.001] and the lower IPFP thickness change ratio [OR = 0.895, *P* < 0.001] were independent factors associated with AKP after MPFLR (Table 6). The correlation between the three pain-related scores (the Kujala, NRS, and VAS scores) and the two independent factors was showed that the IPFP preservation ratio and the IPFP thickness change ratio were significantly correlated with these pain-related scores [|r| > 0.50, *P* < 0.01] (Table 7).

**Table 6:** Multifactorial logistic regression analysis of factors associated with AKP after MPFLR

Variable	Estimate	Standard Error	OR	95% CI	*P* value
BMI	0.666	0.128	1.946	1.516 to 2.499	n.s
The IPFP thickness	−0.105	0.040	0.901	0.821 to 0.984	n.s
The IPFP preservation ratio	−0.945	0.193	0.389	0.266 to 0.567	< 0.001
The IPFP thickness change ratio	−0.111	0.021	0.895	0.869 to 0.932	< 0.001

*OR* odds ratio, *CI* confidence interval, *BMI* body mass index, *IPFP* infrapatellar fat pad, *n.s.* non-significant

**Table 7:** Correlation coefficients between independent factors and pain-related scores

Variable	The IPFP preservation ratio	The IPFP thickness change ratio	Kujala score	NRS score	VAS score
The IPFP preservation ratio	-	0.556^**^	0.718^**^	−0.703^**^	−0.735^**^
The IPFP thickness change ratio		-	0.671^**^	−0.662^**^	−0.593^**^
Kujala score			-	−0.792^**^	−0.755^**^
NRS score				-	0.738^**^
VAS score					-

*NRS* Numerical Rating Scale, *VAS* Visual Analog Scale, *BMI* body mass index, *IPFP* infrapatellar fat pad, *n.s.* non-significant, **: *P* < 0.01

## Discussion

The most important finding of this study was that the lower IPFP preservation ratio and the lower IPFP thickness change ratio were independent factors associated with AKP following MPFLR. Additionally, the results showed significant correlations between the three pain-related scores and the independent factors associated with AKP.

### Role of IPFP in knee function

The IPFP is one of the four fat pads surrounding the knee and plays various roles in the knee, such as serving as a vascular supply, providing cushion for the patellar tendon, and secreting inflammatory factors [36, 37]. The IPFP potentially has a significant impact on pain perception because of the presence of nerve branches from the femoral, saphenous, obturator, and sciatic nerves that traverse through it [38]. Compared to other areas, the posterior section of the IPFP and adjacent synovial tissue have a richer blood supply and higher levels of substance P-positive neurons, which induce pain and could potentially influence the occurrence of AKP [39, 40].

AKP, a common and challenging complication post-knee surgery, can hinder postoperative recovery. Yet, there is scarce research on functional and clinical outcomes in patients with AKP after MPFLR. Our findings suggested that regardless of AKP, MPFLR consistently enhances subjective clinical outcomes. While the Tegner Activity Score, utilized for evaluating exercise and activity levels, was lower in the AKP group compared to the control group. Additionally, there was no significant postoperative change observed in the AKP group compared to their preoperative Tegner scores. Postoperative AKP may also be the reason why patients are not satisfied with the effects of surgery. Early rehabilitation plays a crucial role in ensuring patients achieve favorable outcomes. By identifying the underlying causes of AKP following MPFLR, clinicians can develop more effective interventions and personalized rehabilitation strategies. This targeted approach holds the potential to benefit individuals seeking to resume physical activities by addressing AKP more effectively. This underscores the significance of the research conducted.

### IPFP characteristics and AKP development

The IPFP is a dynamic and mobile structure that deforms during knee motion [41, 42]. US evaluations of the IPFP thickness have shown utility in studies of patients post-ACL reconstruction and those with knee OA [15, 20]. Studies have demonstrated that the superficial IPFP layer presented greater elasticity than the deeper one [43]. Recently, Kitagawa T et al. [31] reported that the thickness change ratio of the superficial part of the IPFP during knee flexion was lower in reconstructed knees than in contralateral knees after ACL reconstruction. They also demonstrated that the lower thickness change ratio was related to AKP [32]. A decreased ratio of change in IPFP measured with US had a negative impact on pain and lower extremity motor function in deep flexion [15, 16], which was consistent with the findings. This may be due to the decrease in dynamics of the IPFP, especially in the superficial part of the IPFP, which may cause IPFP impingement or change the pressure of the infrapatellar tissue.

Several factors, including the IPFP size, have been reported to affect AKP [25, 44]. In this study, the IPFP preservation ratio was lower in the AKP group compared to the control group. Synovitis after knee trauma or surgery can induce inflammation and hypertrophy of the infrapatellar fat pad [20]. Mechanical impact of soft tissue is common [18], which often brings discomfort and pain to patients. The results may be the opposite. Following MPFLR, AKP patients experienced a greater decrease in the IPFP volume. Wallace, Kyle G et al. [16] found the volume of IPFP assessed using MRI was previously reported to be lower on the reconstructed side than on the non-reconstructed side. In addition, greater IPFP volumes may play a role in long-term joint health. Evidence is emerging that the IPFP functions as an exocrine tissue that contributes to the metabolic processes at the joint [36, 45]. However, it is unclear whether the exocrine role is protective or causes joint tissue degradation and the mechanism of the lower IPFP preservation ratio in AKP patients after MPFLR is still unclear. Therefore, future studies should focus on the role of the IPFP in the occurrence of AKP and whether the change of the IPFP volume after MPFLR indicates biological changes related to joint tissue metabolism.

Although BMI and the IPFP thickness were not independent factors associated with AKP, there were differences between the two groups. Studies have shown that AKP patients have higher BMI [46], which is consistent with the findings. BMI has also been shown to be a risk factor associated with poor outcomes after MPFLR [47]. For both age and gender, no significant difference was detected between the two groups, aligning with the conclusions drawn by Migliorini et al. [48]. The IPFP is pivotal in knee metabolism and inflammation, secreting various cytokines [49, 50]. Alterations in IPFP thickness and morphology can impact adipose tissue cell numbers, influencing inflammatory cytokine levels and potentially leading to joint tissue pathology [33]. Consequently, changes in IPFP thickness may be significant in AKP among postoperative patients. However, multifactorial analysis revealed they were not independent factors. Further studies are needed to clarify the relationship and explore its specific mechanism.

### Clinical implications and future directions

This study was the first to show an association between the IPFP and AKP after double-bundle anatomic MPFLR. Real-time information may be obtained in the clinical setting by using US to evaluate IPFP movement against AKP, which may provide a better diagnostic approach. Furthermore, information obtained from non-invasive US and MRI evaluations of AKP after MPFLR can be shared with patients.

Feller et al. [51] reported favorable clinical outcomes but noted a 38% incidence of AKP with isolated MPFLR for recurrent PD. In contrast, the AKP incidence in patients after MPFLR in this study was 20.4%. The variance in AKP incidence may arise from the absence of a standardized definition. Researchers often relied on their own criteria and diverse assessment tools [52, 53], including the Kujala score, visual analog scale, and numerical analogue scale, to diagnose AKP. However, to address this inconsistency, our criteria include a variety of indicators, and the judgment of AKP was stricter and more comprehensive, which also made the results more accurate. In addition, this study also found that the IPFP preservation ratio and the IPFP thickness change ratio, two independent factors related to AKP, were significantly correlated with the Kujala, VAS and NRS pain-related scores. This provided additional evidence that AKP was closely related to both the IPFP preservation ratio and the IPFP thickness change ratio after MPFLR.

### Limitations

This study has several limitations. First, this retrospective study had limitations inherent to all retrospective studies. However, it still revealed new information about the knee joint changes of AKP after MPFLR and tested MRI and US as possible evaluation tools. Second, there was an unequal proportion of female and male patients, while the prevalence of PD was higher in the female than the male population. Third, given that some data were derived through self-report measurements, this may present accuracy and reliability issues [54]. Forth, this study did not assess patellar tilt, which may have an impact on the results and might restrict the generalizability of our findings. In future studies, bone pathologies should be strictly evaluated to make the results more accurate. Fifth, the limitation is the variability in infrapatellar fat pad tissue volume among individuals. Since this varies personally, generalizing findings may be challenging. Additionally, not comparing infrapatellar fat pad tissue differences between the operated and contralateral knees postoperatively is an oversight. Finally, the current research only included the changes of IPFP under non-weight-bearing conditions, so future research should consider the evaluation of the IPFP under weight-bearing conditions. And further studies designed to examine quantitative and qualitative changes in the IPFP and their impact on AKP with a larger sample size are needed. In future studies, researchers will investigate changes over the course of time, or improvements by way of intervention, using a longitudinal design.

## Conclusion

The study revealed that patients after MPFLR in the AKP group had larger BMI, smaller IPFP thickness, and lower IPFP change ratio and preservation ratio. The lower IPFP change ratio and the lower IPFP preservation ratio might emerge as independent factors linked to AKP following MPFLR. Early detection and targeted intervention of the underlying pain sources can pave the way for tailored rehabilitation programs and improved surgical outcomes.

## Data Availability

The data used or analyzed during the current study are available from the corresponding author on reasonable request.

## Abbreviations

PD: Patellar dislocation
MPFL: Medial patellofemoral ligament
MPFLR: Medial patellofemoral ligament reconstruction
IPFP: Infrapatellar fat pad
AKP: Anterior knee pain
OA: Osteoarthritis
ACL: Anterior cruciate ligament
BMI: Body mass index
NRS: Numerical Rating Scale
VAS: Visual Analog Scale
MRI: Magnetic resonance imaging
US: Ultrasound
ICC: Intraclass correlation coefficient
